# YOLO-Dynamic: A Detection Algorithm for Spaceborne Dynamic Objects

**DOI:** 10.3390/s24237684

**Published:** 2024-11-30

**Authors:** Haiying Zhang, Zhengyang Li, Chunyan Wang

**Affiliations:** 1 Opto-Electronics Engineering College, Changchun University of Science and Technology, Changchun 130022, China; zhyhyds@outlook.com; 2 Nanjing Institute of Astronomical Optics & Technology, Nanjing 210042, China; zyli@niaot.ac.cn

**Keywords:** spaceborne dynamic object detection, YOLOv8, SC_Block_C2f, LASF_Neck, multi-scale feature fusion

## Abstract

Ground-based detection of spaceborne dynamic objects, such as near-Earth asteroids and space debris, is essential for ensuring the safety of space operations. This paper presents YOLO-Dynamic, a novel detection algorithm aimed at addressing the limitations of existing models, particularly in complex environments and small-object detection. The proposed algorithm introduces two newly designed modules: the SC_Block_C2f and the LASF_Neck. SC_Block_C2f, developed in this study, integrates StarNet and Convolutional Gated Linear Unit (CGLU) operations, improving small-object recognition and feature extraction. Meanwhile, LASF_Neck employs a lightweight multi-scale architecture for optimized feature fusion and faster detection. The YOLO-Dynamic algorithm’s performance was validated on real-world images captured at Antarctic observatory sites. Compared to the baseline YOLOv8s model, YOLO-Dynamic achieved a 7% increase in mAP@0.5 and a 10.3% improvement in mAP@0.5:0.95. Additionally, the number of parameters was reduced by 1.48 M, and floating-point operations decreased by 3.8 G. These results confirm that YOLO-Dynamic not only delivers superior detection accuracy but also maintains computational efficiency, making it well suited for real-world applications requiring reliable and efficient spaceborne object detection.

## 1. Introduction

With the increasing frequency of space activities, it is crucial to protect in-orbit satellites and facilities, such as the International Space Station, from threats posed by spaceborne moving objects. Efficient and accurate detection of these objects not only determines their orbits and physical characteristics but also provides reliable data support for early warning systems, ensuring spacecraft safety.

Antarctic observatory sites, with their unique advantages such as prolonged polar nights and minimal atmospheric interference, offer superior conditions for astronomical observations [1]. However, given the harsh polar environment and the absence of on-site personnel, the real-time nature of space object detection demands the use of real-time detection algorithms. These algorithms are essential for accurately identifying moving space objects and processing detection data in real time, enabling efficient, continuous observation and early warning without human intervention.

Typical methods for detecting spaceborne moving objects include multi-sensor joint positioning [2], joint detection and tracking using Kalman filtering [3], developing specialized processing channels for different imaging scenarios [4], and employing image differencing with multiple hypothesis tracking [5]. While these approaches each offer unique advantages, challenges remain in terms of detection accuracy and computational efficiency.

In machine learning-based methods, random forests [6] provide higher accuracy, while support vector machines (SVMs) [7] are more suitable for classification tasks. In recent years, deep learning techniques have been increasingly applied to the detection and tracking of spaceborne moving objects [8]. Examples include using U-Net to extract the trajectories of these objects [9], improving detection accuracy through modified SDebrisNet, and combining YOLO with U-Net for enhanced performance [10].

The use of YOLO (You Only Look Once) for detecting spaceborne moving objects aligns with scenarios that require real-time monitoring, rapid response, and unattended operations. The YOLO series algorithms represent a significant breakthrough in deep learning, achieving remarkable results in object detection and gaining widespread application across various domains. Since its initial release, successive versions of YOLO have introduced improvements in model architecture, feature extraction, and fusion, resulting in enhanced detection accuracy and computational efficiency. Current advancements focus on model lightweighting, feature enhancement, and real-time optimization.

To further improve detection accuracy and computational efficiency for spaceborne moving objects, this paper proposes the YOLO-Dynamic algorithm. The algorithm introduces two novel components: the lightweight SC_block module and the newly designed LASF_Neck network structure. The SC_Block module, designed in this study, integrates StarNet and CGLU technologies. This combination optimizes feature extraction and processing, significantly enhancing the model’s ability to recognize spaceborne dynamic objects. Meanwhile, the LASF_Neck structure improves detection accuracy and efficiency through multi-scale feature fusion and enhancement. Ablation experiments and comparisons with mainstream algorithms demonstrate the effectiveness of the proposed improvements.

## 2. Related Work

Space dynamic object detection tasks are a part of the field of computer vision, aiming to identify and locate specific categories of target objects in images or videos. Specifically, object detection requires the system to recognize the targets present in an image and accurately annotate their positions, usually represented by bounding boxes. This task requires models not only to classify the object categories in the image but also to precisely determine their positional coordinates within the image. In earlier years, traditional object detection mainly relied on manually designed features and conventional machine learning algorithms.

The Viola–Jones detector [11], proposed by Paul Viola and Michael Jones in 2001, is a classic real-time object detection method primarily used for face detection but also suitable for other objects. This detector employs Haar-like features and the Adaboost algorithm to train strong classifiers and utilizes cascade structures and sliding window techniques to achieve fast object detection. Its unique design allows for the rapid elimination of negative examples in the early stages, improving overall detection speed. In 2005, N. Dalal and B. Triggs proposed the HOG algorithm [12], a feature extraction method for object detection. By computing histograms of oriented gradients in local regions of the image, HOG captures the texture and shape information of targets, laying the foundation for subsequent pedestrian detection algorithms. However, its drawbacks include high sensitivity to noise and poor performance in handling scale variations. In 2008, Deformable Part Models (DPM) were proposed [13]. The core principle is to decompose the target object into multiple parts and then build corresponding discriminative models for each part. These part models can adapt to deformations of the target object, enhancing the robustness of object detection. With the development of deep learning, increased computational power, and diversified data acquisition methods, convolutional neural networks have gradually become the mainstay of object detection.

In 2012, Alex Krizhevsky and others proposed AlexNet [14], which mainly adopted a deep neural network architecture, ReLU activation functions, and dropout techniques, significantly reducing the model’s error rate on the test set. It won the championship in the same year’s ImageNet Large Scale Visual Recognition Challenge (ILSVRC) [15]. Since then, convolutional neural networks have rapidly gained fame, becoming a focus for many scholars. In 2014, the research team from the Visual Geometry Group at Oxford University proposed VGGNet [16], which used a relatively simple and regular structure while increasing the network depth, significantly reducing the error rate in image classification. However, this network consumed more computational resources and faced issues like gradient explosion. In the same year, Girshick and others [17] proposed the Region-based Convolutional Neural Network (R-CNN) model at the 2014 CVPR conference. The model mainly includes the following steps: after inputting an image, candidate regions are determined; these candidate regions are sent into a neural network for feature extraction; the extracted features are then classified using a support vector machine; and finally, the prediction results of the targets are output. This model performed remarkably in object detection tasks and stood out on the Pascal VOC 2007 dataset [18]. However, R-CNN requires input images and candidate regions to be of consistent size, limiting the flexibility of the network model.

In 2015, Kaiming He and others [19] introduced ResNet, successfully solving the problem of vanishing gradients through a cleverly designed residual structure, winning the championship in that year’s LSVRC competition. In the same year, Fast R-CNN [20] was proposed as an improved object detection model based on R-CNN. Compared to R-CNN, Fast R-CNN adopted the Region of Interest (ROI) pooling technique, resulting in lower model complexity and faster training speed. The following year, Faster R-CNN [21] was proposed, introducing two innovations compared to Fast R-CNN: the concept of anchor boxes and the Region Proposal Network (RPN) technology. In the same year, Joseph Redmon and others [22] collaborated to propose the YOLO (You Only Look Once) algorithm, whose innovation lies in transforming the object detection task into a regression problem. Compared to traditional two-stage detection methods, YOLO divides the image into grids and then performs regression prediction independently for each grid. The core idea of this method is to directly output the bounding boxes and category information of objects through a neural network, making the entire object detection process more efficient while maintaining low inference latency. In the same year, the SSD network [23], which borrowed ideas from YOLO and Faster R-CNN algorithms, was proposed. This model can extract effective information when processing feature maps of different sizes and dimensions and achieves object detection through regression prediction, outperforming YOLO.

In 2017, the emergence of the Feature Pyramid Network (FPN) structure [24] allowed networks to utilize feature information from different levels by constructing a pyramid-shaped feature hierarchy, enabling models to effectively detect small objects. In the same year, Joseph Redmon and others [25] proposed YOLO9000 based on YOLO, improving the model’s generality and performance through batch normalization and anchor boxes. Meanwhile, researchers like Lin et al. [26] proposed the RetinaNet model. This innovative model introduced Focal Loss to address the problem of class imbalance between positive and negative samples in object detection. At the same time, RetinaNet uses the FPN structure, making the network more capable of multi-scale perception, allowing RetinaNet to achieve significant performance improvements in handling class imbalance and multi-scale object detection.

In 2018, Joseph Redmon continued to develop YOLOv3 [27] based on YOLOv2. YOLOv3 uses the Darknet-53 backbone network and employs multi-label classification, significantly improving detection accuracy. In 2020, researchers like Alexey Bochkovskiy [28] proposed the YOLOv4 algorithm model based on YOLOv3. By using a series of strategies such as data augmentation, label smoothing [29], CIOU loss [30], SPP [31], CSPNet [32], and PaNet [33], it achieved better accuracy and faster speed. Subsequently, Ultralytics released the YOLOv5 algorithm model, which has undergone upgrades and iterations. In the same year, Megvii Technology released the YOLOX model [34], improving algorithm accuracy by integrating strategies such as decoupled heads, SimOTA, and anchor-free methods. In 2022, the team led by Alexey Bochkovskiy [35] proposed the YOLOv7 algorithm, further enhancing the network’s accuracy and speed by using strategies like Efficient Aggregation Networks, dynamic label assignment, and re-parameterized convolution.

In the field of deep learning, spatial dynamic object detection is a research area that has received widespread attention, with numerous outstanding algorithm models emerging every year. Some research teams focus on designing innovative object detection networks, dedicated to exploring new architectures and methods. Other researchers draw advanced ideas from different fields to improve existing models, enhancing the speed and accuracy of object detection. Meanwhile, some scholars apply object detection technology to practical problems, promoting the development of these technologies.

## 3. Antarctic Observational Conditions

Astronomical observations using telescopes are often constrained by atmospheric turbulence and sky background interference. However, Antarctica offers unique advantages in terms of climate conditions and observation periods compared to mid- and low-latitude observatory sites. Kunlun Station, located at Dome A, is situated at an average altitude of over 4000 m. The atmospheric turbulence layer at this site is only about 14 m thick, with a median seeing of approximately 0.3 arcseconds, offering exceptional conditions for astronomical observations [36]. During nighttime observations, when the telescope is raised 8 m above the ground, free-atmosphere seeing can be achieved 31% of the time. When raised to 14 m, this increases to nearly 50% [37]. The polar night at Dome A lasts for nearly five months, with over 90% of nights being clear [38].

During the daytime, the sky background at Kunlun Station is 2–3 magnitudes darker in the red visible and near-infrared bands compared to conventional observatory sites [1]. This allows for the detection of large spaceborne moving objects even under backlit conditions. Additionally, near-Earth space debris has a higher transit frequency over the polar regions. Polar-orbiting debris accounts for 55% of all space debris, with every orbit passing over Kunlun Station. Furthermore, 82.6% of all debris orbits pass over Kunlun Station, and 93.7% of the debris passes over the station for more than half of its orbits [1]. These characteristics make Antarctic observatories superior in terms of observational efficiency and coverage compared to other sites.

The Multi-band Survey Telescope (MST), designed for time-domain surveys, was successfully installed at Zhongshan Station in Antarctica in 2022. It is the first telescope in Antarctica capable of simultaneous multi-band observations. The MST consists of four Newtonian telescopes, each with a 150 mm aperture. Two of these telescopes are equipped with motorized filter wheels, enabling simultaneous observations in three different bands (L, R, G, and B). The MST is mounted on a high-precision equatorial mount and features an indium tin oxide (ITO) anti-frost heated window to ensure reliable operation under extreme cold and snowy conditions [39].

In the future, the MST will be deployed at multiple Antarctic observatory sites, including Kunlun Station, to leverage Antarctica’s favorable conditions for multi-band synchronized observations. These efforts will facilitate exoplanet detection, astrometry, and time-domain surveys, while also laying the groundwork for future space debris monitoring and the development of larger-scale astronomical observation arrays.

## 4. Algorithm Principles and Improvements

This paper proposes YOLO-Dynamic, a model based on the YOLOv8s series, tailored for spaceborne dynamic object detection. The proposed model enhances detection accuracy while further reducing computational complexity and the number of parameters.

### 4.1. YOLOv8 Model

**Figure 1:**
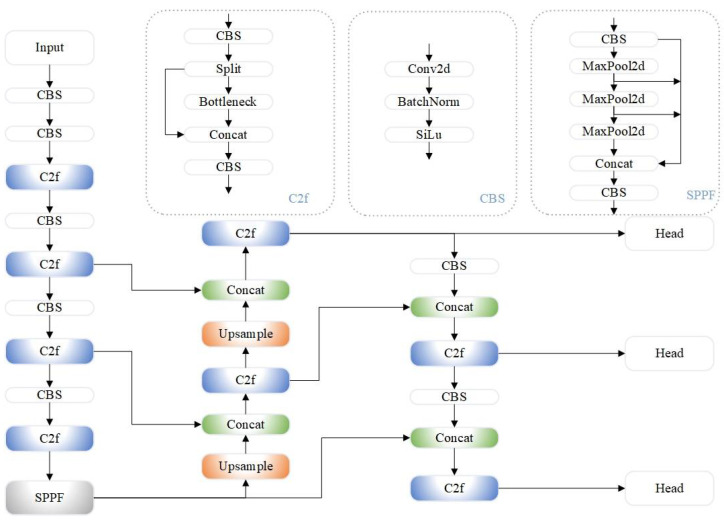
YOLOv8 network architecture.

The architecture of the YOLOv8 model can be divided into four parts: the Input Layer, Backbone Network, Neck, and Detection Head. The YOLOv8 network model is shown in Figure 1.

- Input Layer:

The input layer adjusts the dimensions of the input images to meet training requirements. It also performs operations such as scaling, tone adjustments, and Mosaic data augmentation to enhance the diversity of the training data.

- Backbone Network:

The backbone network adopts a Cross-Stage Partial Network (CSPNet) architecture, which is designed to enhance feature extraction capabilities. It improves overall model performance by balancing computational efficiency and feature representation capacity.

- Neck:

The neck is responsible for feature fusion and is designed using a combination of the Feature Pyramid Network (FPN) and Path Aggregation Network (PAN). These networks integrate feature information from multiple scales, improving the model’s ability to detect objects at different sizes and positions.

- Detection Head:

The detection head employs a decoupled head structure, separating the detection and classification tasks. This separation reduces the number of parameters and the complexity of the model, thereby enhancing its generalization ability and robustness. The detection head generates confidence scores and position information for different objects, achieving accurate object detection.

### 4.2. YOLO-Dynamic

**Figure 2:**
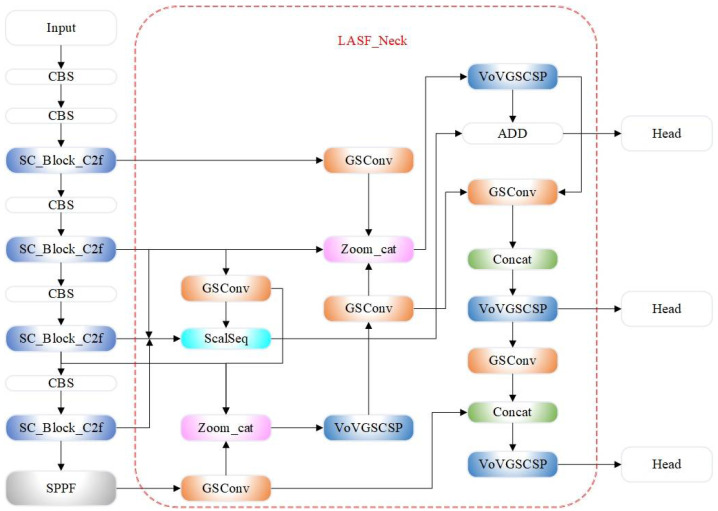
YOLO-Dynamic algorithm architecture.

Building on the YOLOv8s algorithm model, this paper introduces the SC_Block_C2f structure and the LASF_Neck network structure to form a novel detection model called YOLO-Dynamic, designed for spaceborne dynamic object detection. The proposed algorithm maintains a lightweight design while effectively enhancing feature extraction and small-object detection capabilities. As shown in Figure 2, the YOLO-Dynamic architecture consists of three main components: Backbone, LASF_Neck, and Head.

In the Backbone section, the input image passes through multiple layers of conditional convolution and the SC_Block_C2f module, where features are extracted layer by layer. These features are further processed by the Spatial Pyramid Pooling-Fast (SPPF) module to enhance the receptive field. Once the Backbone processing is complete, the feature maps are passed to the Section 4.2.2.

The LASF_Neck integrates and enhances features using modules such as GSConv, ScalSeq, and Zoom_cat. Feature maps are fused across different scales through operations like addition and concatenation. The VoV-GSCSP module is employed to further extract and fuse features before passing them to the Detection Head, where object detection is performed.

Throughout the architecture, the specific connections between modules ensure the effective transmission and processing of information, significantly improving the detection accuracy and precision of the YOLO-Dynamic model.

#### 4.2.1. Backbone Network Improvement

This paper introduces a new lightweight structure called SC_Block_C2f by combining the StarNet network [40], Convolutional Gated Linear Unit (CGLU) [41], and C2f structure.

**Figure 3:**
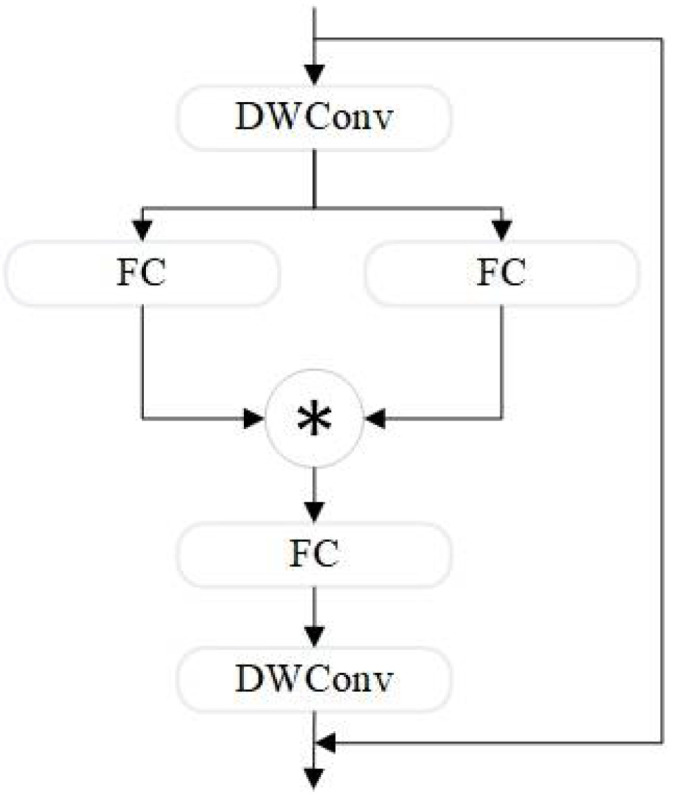
Star_Block architecture. "✳" represents element-wise multiplication.

The StarNet_Block structure, shown in Figure 3, is based on the “star operation”, which enhances feature representation by mapping inputs to high-dimensional, non-linear feature spaces through element-wise multiplication.

**Figure 4:**
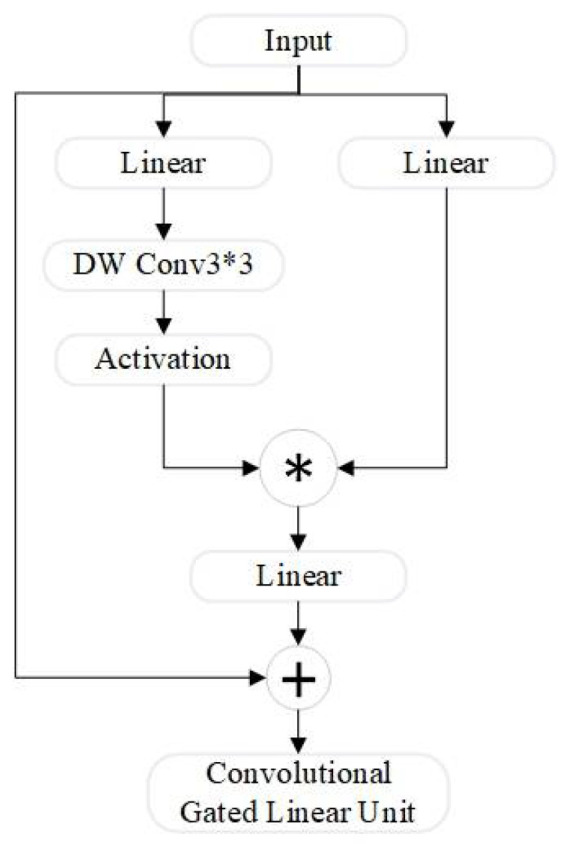
CGLU architecture.

The CGLU structure, compared to the baseline YOLOv8s model YOLO-Dynamic in Figure 4, consists of two linear projections combined through element-wise multiplication, forming a gated channel attention mechanism based on neighboring features. The advantage of CGLU is that it generates unique gating signals for each token based on fine-grained neighboring features, addressing the coarse granularity problem associated with global average pooling.

**Figure 5:**
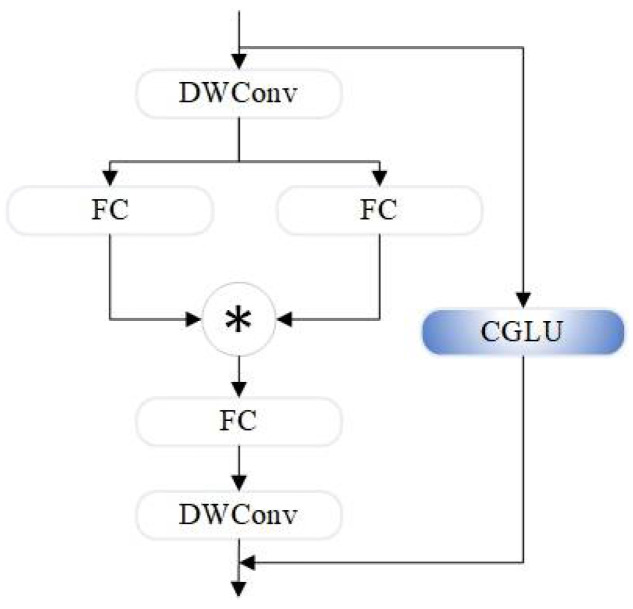
SC_Block architecture.

The novel SC_Block structure, depicted in Figure 5, introduces a conditional gating mechanism, enabling the network to dynamically adjust the information flow according to different input features. This design retains the lightweight characteristics of the structure while significantly enhancing the model’s ability to handle complex or varying inputs. SC_Block achieves more efficient and precise feature modulation and extraction, further optimizing the overall network performance.

**Figure 6:**
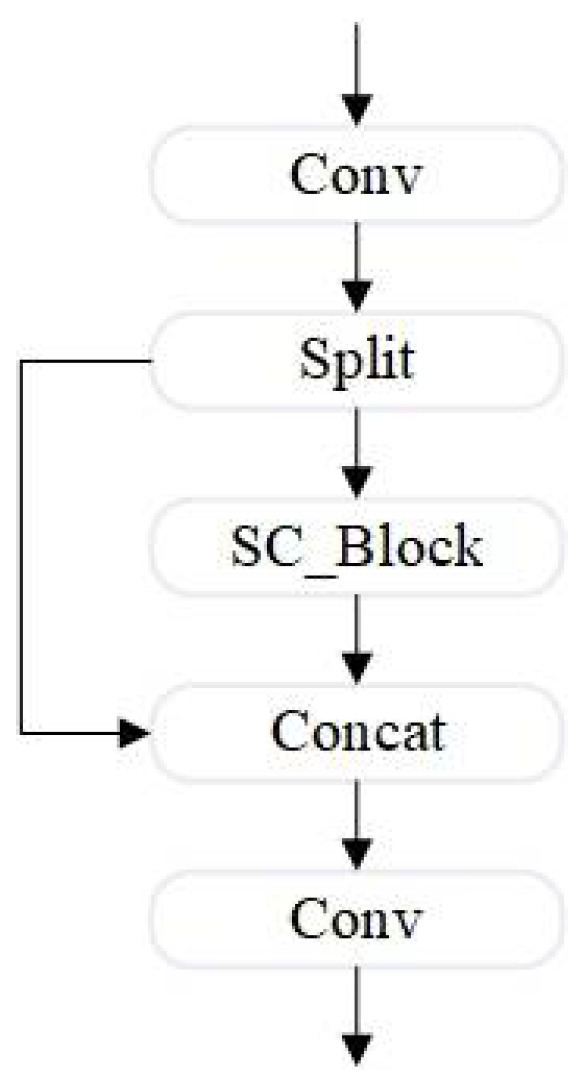
SC_Block_c2f architecture.

The proposed SC_Block is integrated with the C2f structure of YOLOv8s to form the SC_Block_C2f module, as shown in Figure 6. This combined structure maintains a lightweight design while dynamically adjusting to different input features, improving the model’s capability to process complex or changing inputs effectively.

#### 4.2.2. LASF_Neck

This paper presents a novel multi-scale lightweight neck structure called LASF_Neck, inspired by the concepts of SlimNeck [42] and ScalSeq [43]. LASF_Neck enhances feature extraction for complex scenarios and small objects while maintaining a lightweight design.

**Figure 7:**
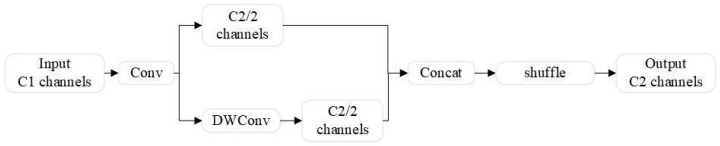
GSConv architecture.

The design goal of SlimNeck is to reduce the computational cost of the model while improving detection accuracy. Its core component is the GSConv module, an innovative lightweight convolution technique [19], which consists of three parts: standard convolution, depthwise separable convolution, and a shuffle operation, as shown in Figure 7.

In the GSConv module, features extracted through standard convolution (Conv) and depthwise convolution (DWConv) are concatenated. A subsequent shuffle operation ensures that the feature information from the Conv layer is fully integrated into the DWConv output, improving feature fusion and representation.

**Figure 8:**
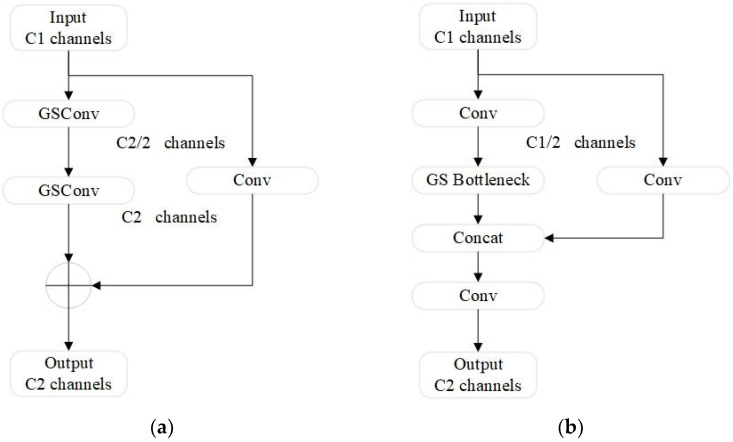
GS Bottleneck and VoV-GSCSP module architectures for enhanced feature processing (**a**) GS bottleneck (left); (**b**) VoV-GSCSP (right).

The VoV-GSCSP module combines the GS bottleneck structure with the CSPNet concept, as shown in Figure 8b. The GS bottleneck consists of two GSConv layers and one standard convolution (Conv) layer, as illustrated in Figure 8a.

As an enhanced module based on GSConv, the GS bottleneck aims to further improve the network’s feature processing capabilities, enhancing the non-linear expression of features and information reuse. The VoV-GSCSP module efficiently aggregates features across stages, improving feature extraction and network performance while maintaining a lightweight structure.

**Figure 9:**
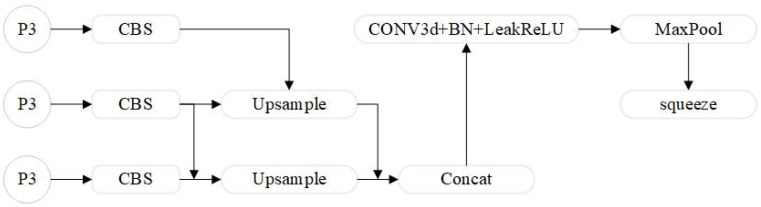
ScalSeq.

ScalSeq [43] is a module specifically designed for processing and fusing multi-scale features, as shown in Figure 9. This module first unifies the channel dimensions of all input feature maps through convolutional layers. It then performs deeper feature fusion through additional convolutional operations.

By incorporating batch normalization and the LeakyReLU activation function, ScalSeq effectively enhances training stability and improves the model’s non-linear expression capabilities. Additionally, the max-pooling operation not only reduces the spatial dimensions of the features, lowering computational complexity but also capturing more critical feature information.

This paper integrates the concepts of ScalSeq and SlimNeck to propose the LASF_Neck network for the neck structure. The LASF_Neck not only optimizes computational efficiency but also enhances the model’s ability to understand complex scenarios by flexibly adjusting and fusing multi-scale features.

## 5. Experiments and Results Analysis

### 5.1. Dataset Preparation

The dataset of moving space objects used in this study originates from telescope observation images at the Antarctic astronomical observatory station. After data augmentation, the dataset contains a total of 4000 images, divided into a training set (2800 images), a validation set (800 images), and a test set (400 images). Since the telescope tracks at stellar speeds, moving targets appear as “short lines” in the images when exposing high-magnitude celestial bodies. These lines, serving as significant features of space objects, are used to train the YOLO network. The targets in the dataset encompass small, medium, and large objects, which aids in the evaluation of subsequent metrics. Specifically, small targets are those smaller than 32 pixels × 32 pixels; medium targets range between 32 pixels × 32 pixels and 96 pixels × 96 pixels; and large targets are those larger than 96 pixels × 96 pixels.

### 5.2. Experimental Setup and Evaluation Metrics

Experimental setup: the hardware configuration used for the experiments is detailed in Table 1.

For the model training, the following parameters were configured:

- Input Image Size: 640 × 640;
- Training Epochs: 300 complete iterations over the training dataset;
- Batch Size: 32 images per batch;
- Data Loading Threads: 16 workers were used to accelerate data reading and preprocessing;
- Optimizer: Stochastic Gradient Descent (SGD).

All experiments were conducted without using pre-trained weights to ensure the purity of the results.

### 5.3. Evaluation Metrics

To objectively evaluate the performance of the CIFM-YOLO algorithm, the following metrics were selected:

- Precision (P);
- Recall (R);
- Mean Average Precision (mAP);
- Intersection over Union (IoU) Threshold mAP (mAP@0.5 and mAP@0.5:0.95);
- Number of Parameters (Params);
- Floating Point Operations (FLOPs);
- Frames Per Second(FPS).

Here, mAP@0.5 refers to the mean average precision at an IoU threshold of 0.5, while mAP@0.5:0.95 indicates the mean mAP across multiple IoU thresholds ranging from 0.5 to 0.95. The formulas for these metrics are as follows:

(1) $$P=\frac{T_{P}}{T_{P}+F_{P}}\times 100\%,$$

(2) $$R=\frac{T_{P}}{T_{P}+F_{N}}\times 100\%,$$

(3) $$A_{P}=\int_{0}^{1}P(R)dR,$$

(4) $$mAP=\frac{\sum A_{P}}{N_{C}}\times 100\%$$

where, *T_p_* represents the number of correctly detected spaceborne dynamic objects. *F_p_* represents the number of false positives, where non-target objects are incorrectly detected as spaceborne dynamic objects. *F_N_* represents the number of missed spaceborne dynamic objects (false negatives). *A_p_* represents the average precision, calculated based on precision (P) and recall (R). *N_C_* denotes the number of object classes; in this case, N = 1.

FPS (Frames Per Second) represents the number of image frames an algorithm can process per second. A high FPS is crucial for real-time systems because it directly affects how quickly the system responds to environmental changes. A low FPS may lead to delays. Typically, when the FPS reaches 30, it means that real-time detection has been achieved.

#### 5.3.1. Ablation Study

To validate the effectiveness of the proposed YOLO-Dynamic model for spaceborne dynamic object detection, an ablation study was conducted. The original YOLOv8s network and its improved versions with each enhanced module were tested on the test set. The results of the ablation study are shown in Table 2.

Table 2 demonstrates the performance improvements achieved through the ablation study.

- When only the SC_Block_C2f structure is added, the model’s mAP@0.5 and mAP@0.5:0.95 improve by 2.2% and 8.2%, respectively. At the same time, the number of parameters and FLOPs decrease by 8.7% and 11.3%, respectively.
- On the other hand, when only the proposed LASF_Neck network is adopted, the mAP@0.5 and mAP@0.5:0.95 increase by 3.4% and 8.6%, respectively. The parameters decrease by 7.18%, and the FLOPs are reduced by 7.4%.
- When we simultaneously apply our proposed SC_Block_C2f structure and LASF_Neck network to the YOLOv8s algorithm, forming the YOLO-Dynamic algorithm, the mAP@0.5 and mAP@0.5:0.95 increase by 7% and 10.3%, respectively. Meanwhile, the number of parameters and FLOPs decrease by 13.3% and 13.4%, respectively. Additionally, the FPS improves by 10 compared to the baseline algorithm YOLOv8s, indicating that the YOLO-Dynamic algorithm is superior to YOLOv8s in real-time processing.

The results of the ablation study clearly demonstrate the effectiveness of the proposed algorithm improvements.

**Figure 10:**
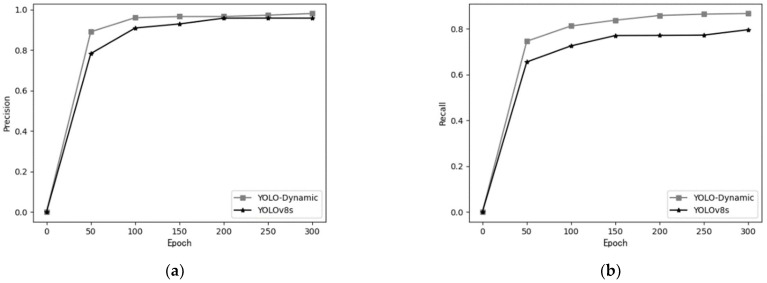
Performance comparison of precision and recall between YOLO-Dynamic and YOLOv8s (**a**) precision; (**b**) recall.

Figure 10 compares the precision and recall between the proposed YOLO-Dynamic algorithm and YOLOv8s. The comparison is made as the number of training epochs increases. From the figure, it is evident that the precision and recall curves of YOLO-Dynamic consistently remain above the corresponding curves of YOLOv8s throughout the training process.

When the curves converge, both the precision and recall of YOLO-Dynamic are significantly higher than those of YOLOv8s. This result demonstrates that the YOLO-Dynamic model outperforms YOLOv8s in terms of both training efficiency and overall performance, highlighting the superior capabilities of the proposed algorithm.

#### 5.3.2. Detailed Comparison with the Baseline Network

**Figure 11:**
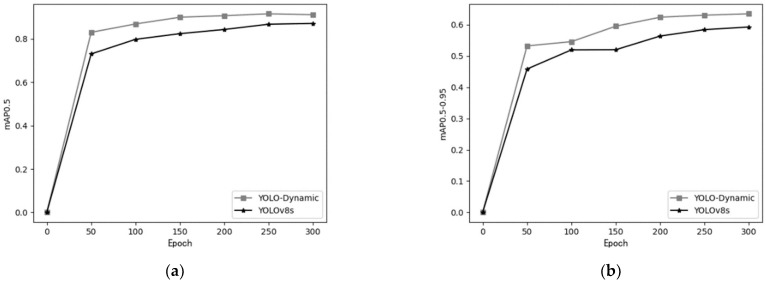
mAP performance comparison between YOLO-Dynamic and YOLOv8s networks (**a**) mAP@0.5; (**b**) mAP@0.5:0.95.

Figure 11 shows the comparison of mAP@0.5 and mAP@0.5:0.95 between the proposed YOLO-Dynamic algorithm and YOLOv8s as the number of epochs increases. The figure demonstrates that YOLO-Dynamic outperforms YOLOv8s across both metrics.

In the left panel, the YOLO-Dynamic curve consistently remains above the YOLOv8s curve as the number of training epochs increases. In the right panel, YOLO-Dynamic exhibits higher detection accuracy at different stages of training, maintaining a clear advantage over YOLOv8s.

Overall, YOLO-Dynamic achieves superior detection performance across various IoU thresholds compared to YOLOv8s. Additionally, the faster convergence of YOLO-Dynamic allows it to reach high detection accuracy earlier in the training process.

**Figure 12:**
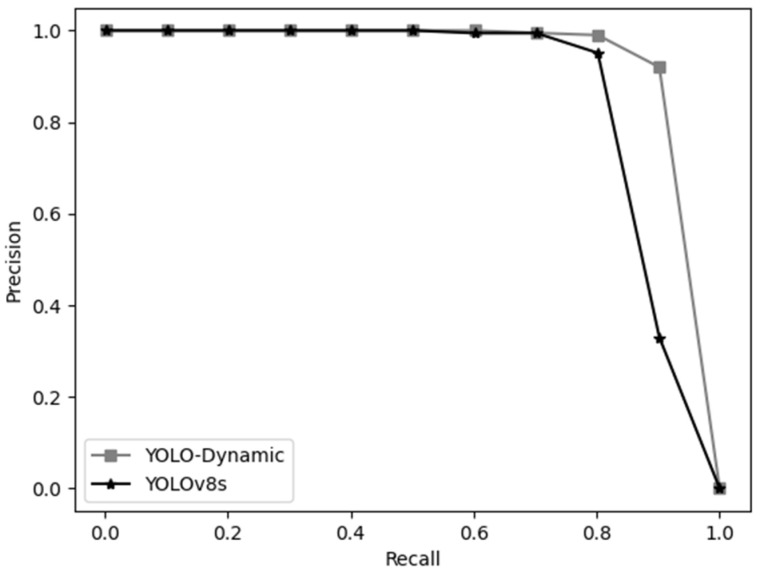
Precision–recall curve comparison between YOLO-Dynamic and YOLOv8s algorithms.

Figure 12 presents the precision–recall comparison between the YOLO-Dynamic and YOLOv8s models. YOLO-Dynamic demonstrates higher precision across most recall levels, particularly excelling in the high recall range (above 0.8). Moreover, the precision–recall curve of YOLO-Dynamic consistently lies above that of YOLOv8s, indicating a superior balance between precision and recall.

Table 3 compares the performance metrics of YOLOv8s and YOLO-Dynamic across different target scales. Specifically, APS, APM, and APL represent the average precision for small, medium, and large objects, respectively, calculated within specific threshold ranges. The results show that the YOLO-Dynamic algorithm achieves higher average precision across all scales compared to YOLOv8s, with improvements of 6.8% for small objects, 3.3% for medium objects, and 9.9% for large objects.

This suggests that YOLO-Dynamic can detect more targets while maintaining high accuracy and stability, confirming its superior performance in handling multiple spaceborne dynamic objects.

#### 5.3.3. Performance Comparison of Different Algorithms

To further validate the detection effectiveness of the proposed YOLO-Dynamic model, a series of detailed comparison experiments were conducted. Several classic models, including Faster R-CNN, YOLOv5s, YOLOv6s, YOLOv7, RT-DETR, YOLOv8s, and YOLO11s were selected as benchmarks to comprehensively evaluate YOLO-Dynamic’s performance across various metrics. The performance comparison of these models is shown in Table 4.

The results presented in Table 4 show that the YOLO-Dynamic algorithm significantly outperforms several classic object detection models across multiple core performance metrics. Specifically, YOLO-Dynamic achieves an F1 score of 95.11%, with mAP@0.5 reaching 94.90% and mAP@0.5:0.95 reaching 64.30%, all of which are clearly superior to other benchmark models.

In addition to accuracy, YOLO-Dynamic demonstrates remarkable efficiency in terms of model parameters and weight size, requiring only 9.65 M parameters and 18.80 MB of weight storage, both much smaller than most classic algorithms.

When compared to the latest YOLO11, YOLO-Dynamic excels in most key metrics. The F1 score of YOLO-Dynamic is 11.52% higher than that of YOLO11, indicating superior accuracy and stability in object detection. Additionally, mAP@0.5 and mAP@0.5:0.95 are improved by 9% and 6.2%, respectively, under varying IoU thresholds, further showcasing YOLO-Dynamic’s outstanding detection capability.

While YOLO-Dynamic requires 9.65 M parameters, slightly more than YOLO11’s 9.41 M, and has a higher floating-point operation count of 24.6 G compared to YOLO11’s 21.3 G, the weight size of YOLO-Dynamic is significantly reduced to 18.8 MB, representing a 48% reduction compared to YOLO11. This reduction in storage cost offsets the slightly higher computational demand, making the model more efficient in practical applications.

In summary, YOLO-Dynamic achieves a better balance between detection accuracy, computational efficiency, and model size. Its superior performance demonstrates enhanced adaptability and robustness in complex dynamic object detection tasks, making it suitable for applications with strict real-time and detection accuracy requirements.

**Figure 13:**
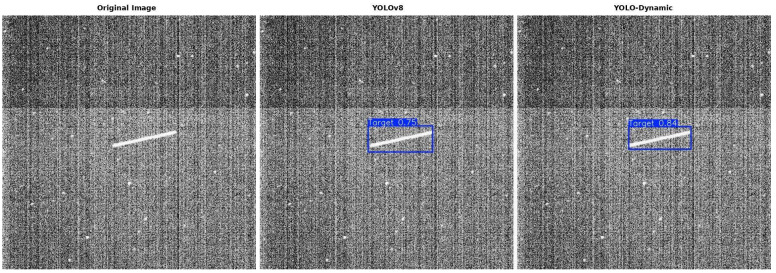
Comparison of results between YOLOv8s and YOLO-Dynamic algorithms.

Figure 13 shows the comparison of detection results between the YOLOv8s algorithm and the proposed YOLO-Dynamic algorithm for spaceborne dynamic object detection. Due to the influence of background or noise, detecting these objects presents certain challenges. However, under typical detection conditions, YOLO-Dynamic demonstrates significantly higher detection accuracy compared to the original YOLOv8s network. The blue boxes in the figure are anchor boxes, and the numbers on the boxes represent the confidence scores.

**Figure 14:**
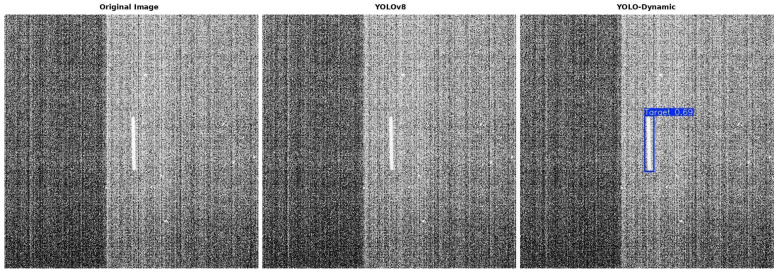
Comparison of results between YOLOv8s and YOLO-Dynamic algorithms.

Figure 14 shows that when the direction of the spaceborne dynamic object’s movement aligns with the impact of the camera’s inherent noise, the original network may fail to detect the object. However, the YOLO-Dynamic algorithm is capable of detecting the object under such conditions to a certain extent.

**Figure 15:**
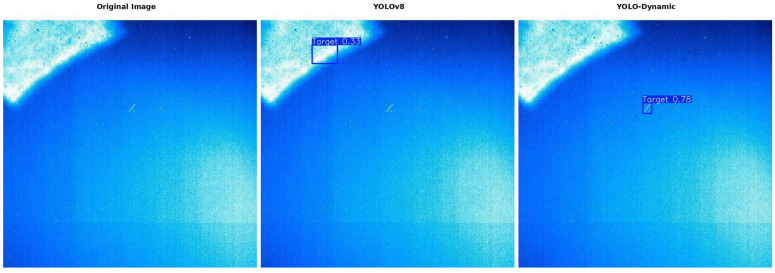
Comparison of results between YOLOv8s and YOLO-Dynamic algorithms.

As shown in Figure 15, for spaceborne dynamic objects, the baseline network YOLOv8s not only fails to detect small targets but also produces false positives, incorrectly identifying a portion of the background as a target. In contrast, our proposed YOLO-Dynamic algorithm successfully detects the correct target. The reason for this difference lies in the fact that the YOLOv8s network primarily focuses on global information, whereas the YOLO-Dynamic network places greater emphasis on the important regions of the image, allowing it to detect the target more accurately. In images with high background complexity, where the target is similar to the background or the noise level is high, YOLO-Dynamic, due to its relatively smaller parameter size compared to larger models, may still experience some misclassifications. However, compared to the baseline model YOLOv8s, YOLO-Dynamic is able to effectively reduce misclassifications in most complex scenarios, demonstrating stronger robustness in dynamic environments and challenging backgrounds.

In addition, for certain bright spots whose features resemble those of spaceborne dynamic objects, YOLO-Dynamic accurately detects the targets, avoiding false detections that could interfere with the detection process.

YOLOv8s shows relatively weaker performance in detecting this type of object. In contrast, the YOLO-Dynamic algorithm exhibits significant advantages in identifying spaceborne dynamic objects, achieving notably higher detection accuracy than YOLOv8s. This comparison not only highlights the efficiency of the YOLO-Dynamic algorithm but also demonstrates the superiority and practicality of the proposed approach.

## 6. Conclusions

Detecting spaceborne dynamic objects is essential for ensuring the safety of space activities. Leveraging the advantages of Antarctic astronomical observations and addressing the real-time requirements of spaceborne object detection, this paper proposes a novel detection algorithm—YOLO-Dynamic—to tackle the challenges of low detection accuracy and poor performance in identifying small objects. This algorithm maintains a lightweight design while significantly enhancing small object detection capability, with its effectiveness validated through a series of experiments.

Firstly, the SC_Block_C2f structure, serving as the backbone network for object detection, effectively captures global features while substantially reducing model complexity and computational load. Secondly, the lightweight multi-scale neck network structure, LASF_Neck, addresses the limitations of the YOLOv8s neck. By integrating and enhancing multi-scale features, LASF_Neck greatly improves the model’s detection accuracy.

Ultimately, the YOLO-Dynamic algorithm achieved a 7% improvement in mAP@0.5 and a 10.3% improvement in mAP@0.5:0.95 over the baseline model. Furthermore, the algorithm reduced the number of parameters by 13.3%, demonstrating its efficiency and effectiveness in spaceborne dynamic object detection. These results highlight the superiority of YOLO-Dynamic in the field of spaceborne dynamic object detection.

## Figures and Tables

**Table 1:** Hardware configuration.

Component	Specification
Operating System	Windows10 (x64)
GPU	NVIDIA GeForce RTX 4090
CPU	Intel Core i9-13900K
Deep Learning Framework	Pytorch 2.1
Programming Environment	Python 3.11, CUDA12.1

**Table 2:** Ablation study results.

Model	SC_Block_C2f	LASF_Neck	P (%)	R (%)	mAP0.5 (%)	mAP0.5–0.95 (%)	Param (M)	Flops (G)	FPS
YOLOv8s	×	×	89.7	82.0	87.9	54.0	11.13	28.4	400
A	√	×	96.6	81.6	90.1	62.2	10.16	25.2	410
B	×	√	94.2	88.3	91.3	62.6	10.33	26.3	400
YOLO-Dynamic	√	√	98.2	92.2	94.9	64.3	9.65	24.6	410

**Table 3:** Comparison of performance metrics between YOLOv8s and YOLO-Dynamic at different target scales.

Model	AP_S_	AP_M_	AP_L_
YOLOv8s	55.4	60.0	35.0
YOLO-Dynamic	62.2	63.3	44.9

**Table 4:** Comparison of mainstream algorithms.

Model	F1(%)	mAP0.5 (%)	mAP0.5–0.95 (%)	Param (M)	Flops (G)	Weight Size (MB)
Faster R-CNN	49.48	57.40	27.10	28.28	X	108.00
SSD	51.24	60.03	30.20	23.88	60.9	20.10
YOLOv5s	86.26	86.20	55.60	7.01	15.8	13.70
YOLOv6s	87.79	88.10	60.20	16.30	44.0	31.30
YOLOv7	87.33	90.20	56.80	36.48	103.2	71.30
RT-DETR	84.16	85.60	55.50	31.99	103.4	63.00
YOLOv8s	85.68	87.90	54.00	11.13	28.4	21.50
YOLO11s	83.59	85.90	58.10	9.41	21.3	36.4
YOLO-Dynamic	95.11	94.90	64.30	9.65	24.6	18.80

## Data Availability

The data presented in this study are available upon request from the corresponding author.

## References

[1] Yang C., Jiang P., Jia M., Zhou X., Zhang S., Zhou H., Li G., Jiang H., Cheng H., Liu J. (2019). Station characteristics and CSTAR data measurement of LEO space-debris monitoring at Kunlun Station, Antarctica. Chin. J. Polar Res..

[2] Liu M., Wang H., Yi H., Xue Y., Wen D., Wang F., Shen Y., Pan Y. (2022). Space debris detection and positioning technology based on multiple star trackers. Appl. Sci..

[3] Su Y., Chen X., Liu G., Cang C., Rao P. (2023). Implementation of Real-Time Space Target Detection and Tracking Algorithm for Space-Based Surveillance. Remote Sens..

[4] Virtanen J., Poikonen J., Säntti T., Komulainen T., Torppa J., Granvik M., Muinonen K., Pentikäinen H., Martikainen J., Näränen J. (2016). Streak detection and analysis pipeline for space-debris optical images. Adv. Space Res..

[5] Sun R., Yu S., Zhao C., Zhang W. (2019). Algorithms for surveying and cataloguing space debris utilizing a wide field of view telescope. Publ. Astron. Soc. Jpn..

[6] Ahmad I., Basheri M., Iqbal M.J., Rahim A. (2018). Performance comparison of support vector machine, random forest, and extreme learning machine for intrusion detection. IEEE Access.

[7] Allworth J., Windrim L., Bennett J., Bryson M. (2021). A transfer learning approach to space debris classification using observational light curve data. Acta Astronaut..

[8] Jharbade P., Dixit M. Detecting space debris using deep learning algorithms: A survey. Proceedings of the 2022 4th International Conference on Inventive Research in Computing Applications (ICIRCA).

[9] De Vittori A., Cipollone R., Di Lizia P., Massari M. (2022). Real-time space object tracklet extraction from telescope survey images with machine learning. Astrodynamics.

[10] Li H., Niu Z., Sun Q., Li Y. (2022). Co-correcting: Combat noisy labels in space debris detection. Remote Sens..

[11] Viola P., Jones M. Rapid object detection using a boosted cascade of simple features. Proceedings of the 2001 IEEE Computer Society Conference on Computer Vision and Pattern Recognition (CVPR 2001).

[12] Dalal N., Triggs B. Histograms of oriented gradients for human detection. Proceedings of the 2005 IEEE Computer Society Conference on Computer Vision and Pattern Recognition.

[13] Felzenszwalb P., McAllester D., Ramanan D. A discriminatively trained, multiscale, deformable part model. Proceedings of the 2008 IEEE Conference on Computer Vision and Pattern Recognition.

[14] Krizhevsky A., Sutskever I., Hinton G.E. (2017). ImageNet classification with deep convolutional neural networks. Commun. ACM.

[15] Russakovsky O., Deng J., Su H., Krause J., Satheesh S., Ma S., Huang Z., Karpathy A., Khosla A., Bernstein M. (2015). Imagenet large scale visual recognition challenge. Int. J. Comput. Vis..

[16] Simonyan K., Zisserman A. (2014). Very deep convolutional networks for large-scale image recognition. arXiv.

[17] Girshick R., Donahue J., Darrell T., Malik J. Rich feature hierarchies for accurate object detection and semantic segmentation. Proceedings of the IEEE Conference on Computer Vision and Pattern Recognition.

[18] Everingham M., Van Gool L., Williams C.K., Winn J., Zisserman A. (2010). The pascal visual object classes (voc) challenge. Int. J. Comput. Vis..

[19] He K., Zhang X., Ren S., Sun J. Deep residual learning for image recognition. Proceedings of the IEEE Conference on Computer Vision and Pattern Recognition.

[20] Girshick R. Fast r-cnn. Proceedings of the IEEE International Conference on Computer Vision.

[21] Ren S., He K., Girshick R., Sun J. Faster r-cnn: Towards real-time object detection with region proposal networks. Proceedings of the Advances in Neural Information Processing Systems.

[22] Redmon J., Divvala S., Girshick R., Farhadi A. You only look once: Unified, real-time object detection. Proceedings of the IEEE Conference on Computer Vision and Pattern Recognition.

[23] Liu W., Anguelov D., Erhan D., Szegedy C., Reed S., Fu C.Y., Berg A.C. (2016). Ssd: Single shot multibox detector. Computer Vision–ECCV 2016: Proceedings of the 14th European Conference, Amsterdam, The Netherlands, 11–14 October 2016, Proceedings, Part I 14.

[24] Lin T.Y., Dollár P., Girshick R., He K., Hariharan B., Belongie S. Feature pyramid networks for object detection. Proceedings of the IEEE Conference on Computer Vision and Pattern Recognition.

[25] Redmon J., Farhadi A. YOLO9000: Better, faster, stronger. Proceedings of the IEEE Conference on Computer Vision and Pattern Recognition.

[26] Lin T.Y., Goyal P., Girshick R., He K., Dollár P. Focal loss for dense object detection. Proceedings of the IEEE International Conference on Computer Vision.

[27] Redmon J., Farhadi A. (2018). Yolov3: An incremental improvement. arXiv.

[28] Bochkovskiy A., Wang C.Y., Liao HY M. (2020). Yolov4: Optimal speed and accuracy of object detection. arXiv.

[29] Müller R., Kornblith S., Hinton G.E. When does label smoothing help? In Proceedings of the Advances in Neural Information Processing Systems, Vancouver, BC, Canada, 8–14 December 2019; Volume 32.

[30] Zheng Z., Wang P., Ren D., Liu W., Ye R., Hu Q., Zuo W. (2021). Enhancing geometric factors in model learning and inference for object detection and instance segmentation. IEEE Trans. Cybern..

[31] He K., Zhang X., Ren S., Sun J. (2015). Spatial pyramid pooling in deep convolutional networks for visual recognition. IEEE Trans. Pattern Anal. Mach. Intell..

[32] Wang C.Y., Liao H.Y., Wu Y.H., Chen P.Y., Hsieh J.W., Yeh I.H. CSPNet: A new backbone that can enhance learning capability of CNN. Proceedings of the IEEE/CVF Conference on Computer Vision and Pattern Recognition Workshops.

[33] Liu S., Qi L., Qin H., Shi J., Jia J. Path aggregation network for instance segmentation. Proceedings of the IEEE Conference on Computer Vision and Pattern Recognition, Salt Lake City.

[34] Ge Z., Liu S., Wang F., Li Z., Sun J. (2021). Yolox: Exceeding yolo series in 2021. arXiv.

[35] Wang C.Y., Bochkovskiy A., Liao H.Y.M. YOLOv7: Trainable bag-of-freebies sets new state-of-the-art for real-time object detectors. Proceedings of the IEEE/CVF Conference on Computer Vision and Pattern Recognition.

[36] Bonner C., Ashley M., Cui X., Feng L., Gong X., Lawrence J., Luong-Van D., Shang Z., Storey J., Wang L. (2010). Thickness of the atmospheric boundary layer above Dome A, Antarctica, during 2009. Publ. Astron. Soc. Pac..

[37] Ma B., Shang Z., Hu Y., Hu K., Wang Y., Yang X., Ashley M.C., Hickson P., Jiang P. (2020). Night-time measurements of astronomical seeing at Dome A in Antarctica. Nature.

[38] Yang Q., Wu X., Wang Z., Hu X., Guo Y., Qing C. (2022). Simulating the night-time astronomical seeing at Dome A using Polar WRF. Mon. Not. R. Astron. Soc..

[39] Chen C., Li Z., Yang C., Han Z., Jiang X., Liu T., Yuan X., Jiang P., Ji T., Yao X. (2023). The Multi-band Survey Telescope at Zhongshan Station, Antarctica. Mon. Not. R. Astron. Soc..

[40] Ma X., Dai X., Bai Y., Wang Y., Fu Y. Rewrite the Stars. Proceedings of the IEEE/CVF Conference on Computer Vision and Pattern Recognition.

[41] Shi D. TransNeXt: Robust Foveal Visual Perception for Vision Transformers. Proceedings of the IEEE/CVF Conference on Computer Vision and Pattern Recognition.

[42] Kang M., Ting C.-M., Ting F.F., Phan R.C.-W. (2024). ASF-YOLO: A novel YOLO model with attentional scale sequence fusion for cell instance segmentation. Image Vis. Comput..

[43] Li H., Li J., Wei H., Liu Z., Zhan Z., Ren Q. (2022). Slim-neck by GSConv: A better design paradigm of detector architectures for autonomous vehicles. arXiv.

